# Culturally responsive recruitment of Black daughter-mother dyads through community engagement

**DOI:** 10.3389/fpubh.2025.1634312

**Published:** 2025-12-10

**Authors:** Tristan Banks, Wrenetha Julion, Shannon Halloway, Spyros Kitsiou, Michael Schoeny, Barbara Swanson, Kashica Webber-Ritchey, Kaitlin Wilhelm, Charleen Yeager, Monique Reed

**Affiliations:** 1 College of Nursing, Rush University, Chicago, IL, United States; 2 College of Nursing, University of Illinois Chicago, Chicago, IL, United States; 3 College of Applied Health Sciences, University of Illinois Chicago, Chicago, IL, United States; 4 College of Science and Health, DePaul University, Chicago, IL, United States

**Keywords:** responsive recruitment, Black Girls, adolescent females, mother-daughter dyads, obesity prevention, culture, race-conscious

## Abstract

**Introduction:**

Black Girls Move is a 12-week, race-conscious, multicomponent, mHealth obesity prevention intervention for Black 7th–10th grade daughters and their mothers. The complex experiences of Black female adolescents and adults necessitate tailored recruitment and retention strategies to address structural, programmatic, and interpersonal barriers to participation. We outline culturally responsive recruitment and retention strategies, lessons learned, and their implications.

**Methods:**

A review of recruitment literature highlighted trust-building as essential. We utilized guidelines for evaluating recruitment feasibility in pilot studies and the Community-Informed Recruitment Plan template of diverse populations as frameworks to assess and refine our recruitment and retention approach.

**Results:**

Key findings included: (1) trust was critical for sustaining participant relationships from screening to baseline, (2) weight eligibility criteria were overly restrictive, (3) recruitment targets needed adjustment to prevent school loss, and (4) competing demands impacted engagement. Refinements involved consulting community leaders and an expert community research consultant, leading to (1) broadening eligibility criteria to include daughters of all weight statuses and 7th–8th graders; (2) increasing incentives to align compensation with time commitments for surveys; and (3) hiring a community health worker to address communication and scheduling issues while fostering trust.

**Discussion:**

Strengthening trust, expanding eligibility, and improving incentives enhanced recruitment and participant engagement. We found this culturally tailored, race-conscious approach was valuable in refining recruitment strategies. Future studies should test the guidelines for evaluating the feasibility of recruitment and the Community-Informed Recruitment Plan template of diverse populations in a large-scale randomized control trial.

## Introduction

Racism is a system that organizes access to opportunities and assigns worth to individuals based on socially constructed perceptions of physical appearance—commonly referred to as race (1). Race is a social categorization rooted in shared physical traits (e.g., White, Black non-Hispanic, Asian, Native American), while ethnicity refers to common cultural or ancestral backgrounds, (e.g., Hispanic/Latino or other ethnicities) (2, 3). Studies have consistently shown that racial and ethnic discrimination contributes to poorer health outcomes (4). For example, Black adolescent females have the highest rates of obesity when compared to their non-Hispanic White counterparts while Hispanic/Latino adolescent females have the second highest rates (5, 6). Obesity in adolescence is identified as >95^th^ percentile of body mass index (BMI) for sex and age (5). While both Black and Hispanic adolescent females experience disproportionate rates of obesity, the contributing factors that influence lifestyle change may differ for these distinct populations. However, there have been few studies of Black adolescent females, limiting the development of culturally appropriate obesity-prevention interventions.

A systematic review of 74 randomized controlled trials (RCTs) focused on obesity prevention among adolescents aged 12 to 18, revealed that of the 31 studies conducted in the United States, Black adolescent females comprised >10% of the participants in only 12 studies (7). These findings underscore the absence of interventions that prioritize the lived experiences of Black adolescent females which is troubling because they have increased risks for type 2 diabetes, cardiovascular disease, asthma, elevated body dissatisfaction, lower self-esteem, ADHD, and depressive symptoms as compared to their racial/ethnic counterparts (8).

Lifestyle modification is a critical component of obesity prevention interventions that target Black adolescent females. In this population, obesity is associated with poor dietary practices and limited physical activity (PA), and complicated by socioeconomic status (6). For example, Black females consume more calories from sugar-sweetened beverages than Black males (9), eat fewer vegetables, and have breakfast less often than recommended compared to other racial/ethnic groups (10). Simultaneously, as they pursue independence and individuality, adolescents choose more sedentary behaviors and become less physically active (11–15). National survey data of adolescents aged 12–15 and 16–19 years old indicate that they do not meet national guidelines for moderate to vigorous PA (16). Alarmingly, Black adolescent females had the lowest step counts and highest rates of sedentary behavior when compared to their peers by gender, race, and age (16). One strategy for promoting a healthy lifestyle in Black adolescent females is to actively involve their mothers in supporting behavior change.

Numerous studies have investigated the role of mothers in shaping their daughters’ health behaviors (17–19). In many Black families, mothers are key contributors to children’s health and social development, often taking the lead in nutrition and caregiving responsibilities (20, 21). Yet despite the potential for mothers to support healthy behaviors of their adolescent daughters, there is a lack of obesity prevention research that includes Black adolescent daughter/mother dyads. To address this gap, our team developed the Black Girls Move (BGM) obesity prevention intervention and is currently conducting a Phase I trial to test its feasibility and preliminary change in primary outcomes. Obesity prevention programs for Black adolescent females must consider that as girls transition into adolescence, they are increasingly influenced by the environmental and developmental factors that affect dietary and PA behaviors which place them at high risk for obesity (6).

### Environmental factors

Some argue health disparities are merely a function of differences in socioeconomic status; however, there is growing evidence that structural racism contributes to racial health disparities (22). Structural racism includes policies and laws which provide advantages to groups based on race while oppressing another race (23, 24). Black adolescent females living in low-income communities are at particular risk for obesity due to racial residential segregation and its role in creating obesogenic environments characterized by reduced access to healthy foods, heavy marketing of unhealthy food (25, 26), few safe, walkable streets, park areas, and exercise facilities (13, 27–30). To prevent obesity and the associated co-morbidities in this at-risk population, programs must acknowledge the impact of racism on dietary and PA behaviors, rather than simply prescribing interventions that do not consider their lived racialized context.

### Developmental factors

Adolescent daughters often rely on their mothers for information, guidance, and support as they work to adopt or maintain healthy behaviors (31). Our cross-sectional, descriptive study to assess the eating behaviors of 10- to 12-year-old Black daughters and their mothers (*N* = 43) showed strong concordance between daughters’ and mothers’ consumption of discretionary calories, such as sugar sweetened beverages (32). Similarly, the influence of mothers in shaping their daughters’ dietary and PA behaviors has been demonstrated repeatedly in cross-sectional studies (6, 25, 33). We developed BGM by acknowledging the environmental and developmental factors that affect Black adolescent females and their mothers. The complex intersection of environmental and developmental factors has exacerbated barriers to the recruitment of Black adolescents into research studies. These recruitment barriers can be structural (transportation, inconvenience) or cultural (lack of cultural concordance of mistrust) and require innovative and tailored recruitment strategies (34). Similarly, Black women face recruitment barriers, such as inability to miss work or lack of trust in researchers (35).

### The need for obesity prevention interventions

A review of randomized controlled trials (RCTs) of culturally tailored obesity treatment interventions (*N* = 44 RCTs) for adolescents aged 12–17 years, found low quality evidence for reducing BMI, inconsistent evidence for improving dietary behaviors, and no evidence for improvements in objective or self-report measures of PA (36). Similar findings were reported in two relevant obesity treatment RCTs Go Girls (37) and the Health Improvement Program (HIP) for teens (38), that tested culturally tailored, family-focused interventions (primarily involving mothers) in overweight and obese Black adolescent females in the US. Go Girls (37) was not effective for reducing adiposity, while the HIP for Teens showed an initial reduction that was not maintained. Several factors may account for the limited effects of these two treatment interventions: (1) once obesity is established in childhood it is hard to reverse (39), which supports our intervention to prevent obesity in Black adolescent females; (2) because Black adolescent females may show greater acceptance of heavier bodies (40, 41), weight maintenance may be a more acceptable goal than weight loss; and (3) parents participated in both interventions, but there was no focus on modifying daughter-mother communication, problem solving, role assignment, or relationship quality.

Adolescence is a critical transitional period marked by growing autonomy, yet it remains a time when parental influence is still essential. It is important to establish relevant recruitment and retention plans for obesity prevention interventions with Black adolescent females. There are known barriers to recruitment of Black adolescents (42), Black mothers, and even Black youth/parent dyads (43). However, less is known about specific barriers to recruitment of Black adolescent daughter/mother dyads. Further, obesity prevention studies with Black daughter/mother dyads to date have not focused on the pivotal developmental period of adolescence. Given the lack of obesity prevention research with Black adolescent daughter and mother dyads, the purpose of this paper is to describe the lessons learned from recruiting participants into the first 2 years of Black Girls Move (BGM), an obesity prevention/weight maintenance intervention.

### Black Girls Move: an obesity prevention/weight maintenance intervention

We designed Black Girls Move (BGM) loosely from the Diabetes Prevention Program, using community engaged research principles of working with communities of interest. We first used interviews and focus groups to draft and refine BGM with Black adolescent daughter/mother dyads and clinical experts (44). We then pilot tested BGM with Black adolescent daughter/mother dyads and incorporated their feedback into its refinement. This iterative process to intervention development produced a culturally tailored and informed intervention (45). Participants provided feedback on placement of content, delivery format, and appropriateness of materials (44). In addition, a culturally acceptable approach in BGM was the concept of *weight maintenance*, which has shown promise as an effective and culturally relevant strategy to address risk for obesity-related diseases in Black females (46). Based on participant feedback, we defined obesity prevention as encompassing both weight loss and weight maintenance.

**Figure 1:**
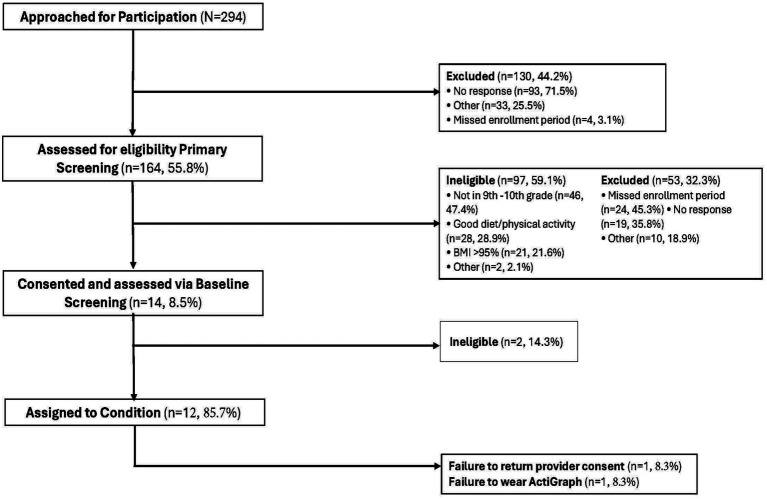
Consort diagram.

BGM is a 12-week race-conscious, multicomponent, mobile health (mHealth) obesity prevention intervention designed for 7^th^–10^th^ grade Black adolescent females and their mothers. Inclusion criteria for daughters were (a) English-speaking; (b) Black; and (c) daily access to the internet outside of school and/or work through an iOS or android smartphone, tablet, or personal computer. Inclusion criteria for mothers were (a) English-speaking; (b) Black; (c) co-residing biological mother or mother-figure and legal guardian of the participating daughter; (d) the person primarily responsible for meals in the household; and (e) access to the internet through an iOS or android smartphone, tablet or personal computer. Originally, we only included daughters that measured as overweight to focus on obesity prevention. However, this criterion excluded 21.6% of daughters (Figure 1). Therefore, we modified our study protocol to include all weight categories with medical release. In addition, the original protocol excluded 7^th^ and 8^th^ graders, which resulted in 47.4% of daughters being ineligible for the study. After speaking with key personnel in schools that served 7^th^–12^th^ grades, we recognized the importance of establishing healthy behaviors in 7^th^ and 8^th^ graders and modified our study protocol to include 7^th^–10^th^ graders. The initial recruitment results enabled us to refine inclusion and exclusion criteria to optimize participant safety, recruit sufficient numbers of participants, and promote generalizability of findings (47). In a future study we plan to include a longer follow-up period of 11^th^ and 12^th^ graders to assess maintenance of behaviors as adolescents transition to post-secondary activities. The original and modified inclusion criteria are further described in Table 1.

**Table 1:** Original and modified inclusion criteria and protocol.

Category	Original daughter inclusion criteria	Modified daughter inclusion criteria
Grade	9 or 10	7–10
Daughter’s weight status		
Underweight <5^th^	Ineligible	Eligible with healthcare provider release^a^
Normal weight 5^th^–85^th^	Only high-normal weight (between ≥50th and <85th percentile for age and gender) eligible with CDC-defined self-reported poor diet (<1 vegetable or <1 fruit per day) OR CDC-defined inadequate PA (< 60 min per day of MVPA, 7 days per week)	All eligible with CDC-defined self-reported poor diet (<1 vegetable or <1 fruit per day) OR CDC-defined inadequate PA (< 60 min per day of MVPA, 7 days per week)
Overweight 85^th^–95^th^	Eligible	Eligible
Obese	Ineligible	Eligible with healthcare provider release^a^

Category	Original protocol	Modified protocol
Sample size	Canceled if 12 dyads per BGM or DOCC condition (*N* = 24) not met	Canceled if *N* = 6 dyads *total* not met. Will run with either BGM or DOCC condition
Consent	In-person	Offered consent and assent via Zoom
Survey administration	In-person via Research Electronic Data Capture (REDCap)	Offered self-administered surveys via REDCap
Baseline, 12-week, 24-week assessment	In-person	Conducted home visits to complete data collection assessments following common home visiting safety guidelines
Frequency of calls	After baseline appointment scheduled, one reminder text made	CHW text check-in every week to remind of upcoming appointment
Incentive	$20 per participant at baseline, 12-week, & 24-week	$40 per participant at baseline, 12-week, & 24-week

a Daughters measured as underweight <5th or obese ≥95th percentile for age and gender will be eligible only with a healthcare provider release to ensure they have established care in the event of an underlying metabolic disorder.

In addition to using mHealth technology, BGM actively engages Black adolescent daughters and their mothers through structured group sessions. The feasibility, preliminary change in outcomes, and impact of BGM will be evaluated against a daughter-only comparison condition (DOCC), which delivers identical content as BGM but excludes maternal participation. In the protocol, eligible participants are randomly assigned 1:1 to either the BGM active treatment or DOCC. BGM is informed by race-conscious, Public Health Critical Race Praxis (PHCRP), Family Systems, and Social Cognitive theories. The intervention development, description of each theoretical component, and outcomes of BGM are described in the BGM protocol (45). The specific aims are to determine in Black adolescent females:

(1) The feasibility (recruitment, retention, and acceptance) of BGM compared to DOCC.; (2) The preliminary change in primary outcomes, including: (a) number of steps per day, and (b) diet quality, measured by the self-reported intake of five components of the Healthy Eating Index-2010 (vegetables, fruit, whole grains, dairy, and protein), as well as secondary outcomes such as: (c) minutes of moderate/vigorous physical activity per week, (d) self-report physical activity, and (e) consumption of sugar sweetened beverages; and (3) The impact of BGM compared to DOCC on differences in theoretical mechanisms of change, including (racial identity, daughter/mother relationship, social cognitions) assessed by self-report measure.

## Materials and methods

To monitor recruitment, we followed the eight-step guidelines for evaluating feasibility of recruitment in pilot studies of diverse populations (48).

### Step 1: describe recruitment goals

As a feasibility study, we did not perform a formal power analysis to determine sample size. Instead, sample size estimates from the Diabetes Prevention Program were used, which suggested at least 5 participants per group to promote peer support, interaction, and group cohesion (49, 50). The group format with dyads provided a mechanism for mothers to share successes and adaptive response strategies with their daughters. Originally, if a minimum of *N* = 12 dyads per school/per condition was not met, the school was canceled but based on the challenges of meeting a total of 24 dyads per school we reduced the cancelation criterion to <*N* = 6 dyads. Six dyads were selected as a minimum recruitment target to account for absences, yet small enough to allow participants time to discuss new foods, ways of preparing foods, or new PA strategies with other dyads.

### Step 2: describe recruitment process

Prior to starting recruitment, we reviewed relevant literature to identify successful culturally tailored recruitment strategies with Black adolescents and determined that building trust, combined with active and passive recruitment strategies, was essential (51–53). Culturally tailored strategies to build trust include community engagement and partnerships, effective communication, culturally competent recruitment materials, involvement of role models, accessibility and convenience, ongoing support, and feedback and adaption (34, 54–56).

Building trust requires a thoughtful and respectful approach that integrates clear communication, cultural sensitivity, and meaningful community engagement. Toward that end, we were intentionally mindful of language nuances that could be ambiguous and lead to unintended connotations. We also cultivated trust by becoming familiar with the community—being present in local spaces, such as connecting with schools and consulting community leaders. We employed team members who shared cultural similarities with participants, and we trained them to interact respectfully with families and school personnel to forge and sustain positive relationships. We conducted in-person enrollment and approached informed consent with care and respect (34, 54–56). Finally, we held in-person meetings with key school leaders to assess the communication and language preferences and norms of students, teachers, and staff.

Active recruitment refers to direct interaction with the target population to increase awareness about the study and provide prospective participants the opportunity to approach the researcher(s) (53, 57, 58). Our active recruitment plan consisted of multiple components. First, we obtained endorsements from the school principals. Second, we built relationships through separate recruitment activities for daughters and mothers. Recruitment activities with daughters occurred during lunchroom or after school activities, while activities with mothers occurred during parent report card pick up. Third, we encouraged daughters to fill out a sheet that collected personal information of daughter (first and last name, grade, and phone number) and mother (first and last name, email, phone number, and preferred time of contact). This information was used to contact mothers to begin the screening process for eligibility. During encounters with both daughters and mothers, a research team member screened for eligibility, explained the study, and scheduled a baseline assessment if time permitted. Otherwise, screening and study explanation were completed on a subsequent telephone call. Lastly, in-person presentations were given to mothers at parent advisory board meetings and to daughters and mothers at a college interest fair. The active recruitment strategies allowed us to obtain contact information for subsequent telephone calls to screen for eligibility. In addition to these strategies, we were responsive to school-specific requests related to relationship building. For example, at two of the high schools, the research team held a health careers seminar during the “flexible period.” At another school, health careers information (59) was presented during the school’s parent night.

Passive recruitment refers to increasing awareness about the study without direct contact (53). Our passive recruitment techniques included distributing flyers through e-mail and posting them in the administrative office, library, and gymnasium of 4 high schools (grades 9–12) and one middle-high school (grades 7–12). The flyer included the Black Girls Move logo, Quick Response (QR) code, information about the study, eligibility criteria, amount of participant gift card compensation for their time, and the principal investigator’s contact information. In addition to trust building, active, and passive recruitment (57, 58) study staff completed training specific to recruitment and retention strategies for minoritized populations. These strategies included training on historical fears of participating in research, partnering with community organizations, and tailoring recruitment materials (60).

### Steps 3 & 4: establish a tracking system and tracking database

Daughter/mother dyads were recruited from five Chicago Public High Schools (one of the five included a middle school). Each high school had a student body of greater than 80% of Black students, poverty rates above 80%, and was not on academic probation. While these criteria limited the number of schools that were eligible to participate in the study, they assured that we recruited from the targeted population. The Consolidated Standards of Reporting Trials (CONSORT) (61), was used to document the flow of participants through the recruitment process. Recruitment data were maintained in a secure Health Insurance Portability and Accountability Act (HIPAA)-compliant database, created in Access for Microsoft 365. The database contained participants’ names, phone numbers, dates of birth, and daughters’ grades and schools. The data manager trained research assistants (RAs) to input data into the Access database using the RA training manual developed by the principal investigator. RAs used the Access database to document the outcome of calls, the number of call attempts, and enter eligibility criteria. RAs followed a documentation protocol for each recruitment activity. For example, lunchroom recruitment protocol included guidelines for RAs to enter participant data into the Access database within 48 h after lunchroom recruitment and to send text messages to all mothers according to the communication protocol. Once contact was made, RAs would describe the study, answer questions, and screen those interested according to the six phases of the communication protocol (Table 2).

**Table 2:** BGM communication protocol.

Phase	Communication attempt
Phase 1	Initial text message within 48 h (two days) of recruitment sign-up.
Phase 2	2^nd^ text message 5 working days of recruitment sign-up.
Phase 3	Initial phone call within 7 working days of recruitment sign-up.
Phase 4	2^nd^ phone call within 10 working days of recruitment sign-up.
Phase 5	3^rd^ and final call within 12 working days of recruitment sign-up.
Phase 6	Document call attempts in Access on the bottom of the participant profile page that has their name, school, etc., and, if no response within 14 working days of recruitment sign-up, fill out the Participant Defer/Withdraw form.

Participant encounter	Sample communication scripts
Wrong number	If the mother’s number was wrong or out of service, RA’s were instructed to make the following text to the daughter: “Please have your mother call/text (number) to join Black Girls Move.”
Study overview	An overview of the study was described as follows: “The Black Girls Move 12-week program is held on Saturday or Sunday mornings at a local park district. The sessions are 90-min, rotating between in-person and virtual via Zoom. You will be required to use your smartphone or tablet. During the intervention participants randomized to BGM receive a Fitbit to self-monitor PA and log into Start Simple with My plate to track diet goals. You will receive text message reminders of your PA and diet goals.”

### Step 5: tracking individual progress

The principal investigator reviewed the CONSORT diagram weekly. Results were shared with co-investigators in weekly meetings to monitor accrual against targets. Enrollment rates were calculated and reported to the funding agency annually. Assessment periods for each school lasted for 8 weeks. The first 4 weeks included lunchroom and after-school recruitment, followed by 4 weeks of baseline screening. Recruitment and enrollment rates for each school were evaluated at the end of each assessment period. Results of recruitment and enrollment were used to plan potential modifications to recruitment methods.

### Steps 6 through 8: summarize recruitment results; summarize feasibility results; utilize tracking data to inform modifications

Two hundred and ninety-four daughters expressed interest and received telephone screening call attempts (Figure 1). Of the 294 dyads, 44.2% (*n* = 130) could not be reached for telephone screening. Of the 164 mothers screened, 59.1% (*n* = 97) were ineligible (i.e., not in 9^th^ or 10^th^ grade, or BMI > 95%) and 32.3% (*n* = 53) were excluded (i.e., missed assessment/enrollment period or no response). Fourteen dyads were consented and assessed for eligibility at baseline screening. Of the 14 dyads that were screened at the in-person baseline screening, 14.3% (*n* = 2) were ineligible for reasons that were not documented and the remaining 12 dyads were assigned to a condition. Of the 12 dyads, one failed to return provider consent and one failed to wear the initial actigraph. Our feasibility data showed that the recruitment goal of 24 dyads per school (12 dyads per condition) was not met, therefore we canceled the intervention at four schools. The last step of the guidelines for evaluating feasibility of recruitment involves modifying recruitment methods based on the tracking data. Our modifications are described below.

## Recruitment and study modifications

### Recruitment modifications

Cunningham-Erves et al. (60), applied a community engaged approach to develop the Community-Informed Recruitment Plan Template, hereafter referred to as, the template. The template serves to help increase recruitment and retention of racial and ethnic minority groups by engaging community members in targeted discussions. The template includes: (1) Recruitment Strategy; (2) A Stakeholder Communication Plan; (3) Evidence of Recruitment Feasibility; (4) Recruitment and Retention Team; (5) Recruitment and Retention Methods; (6) Recruitment and Retention Timeline; (7) Evaluation; and (8) Budget. We applied the template to develop a plan for study modifications.

### Study modifications

Despite our recruitment efforts, we did not meet recruitment targets in Year 1. Hence, we analyzed tracking data, met with community leaders, and engaged an expert community research consultant to modify our recruitment methods. The consultation yielded four lessons that influenced recruitment changes: (1) the importance of maintaining relationships over time to sustain trusting relationships, (2) prior weight eligibility criteria were too prescriptive, (3) the investigators need to change recruitment targets to minimize school loss, and (4) competing demands influenced engagement. Consequently, we (1) hired a community health worker (CHW) to address communication and scheduling concerns and to maintain trusting relationships with eligible participants until the intervention started; (2) amended eligibility criteria to include daughters of all weight status and 7th & 8th graders; and (3) increased incentives to $40 per participant at each data collection point, from $20 per participant to align compensation with the time requested to engage in the baseline assessment process.

#### Maintaining relationships over time to sustain trusting relationships

One lesson learned from Year 1 was the importance of *maintaining* relationships with participants and school partners to *sustain* trust in all research phases. Partnerships within the targeted school communities were initially established prior to receiving study funding and included the schools’ principals, teachers, and nurses. To facilitate a community engaged approach, the principal investigator and project director met with the designated school leaders to explore modifications to our recruitment strategies. These recruitment-focused conversations reinvigorated the initial partnership, leading to robust recommendations that were incorporated into a new recruitment plan tailored for that school. The plan incorporated recommendations on the optimal timing and locations for recruitment and intervention activities. For example, partners from one school recommended the research team connect with the Parent University Program, a monthly meeting for parents and school faculty to discuss students’ career aspirations and extracurricular activities.

In two schools there was an unanticipated three-month gap between the end of study recruitment and the intervention start, resulting in a start date that coincided with the beginning of summer break. School partners advised that participants would have difficulty engaging in the intervention due to changes in their summer school/work schedules, travel, and other summer-related plans. Therefore, the intervention start date was delayed, resulting in some participants losing interest, being unreachable by phone, or aging out of eligibility. After reflection, we identified opportunities to improve subsequent rounds of BGM recruitment and engagement to *sustain* trust once established. First, we scheduled recruitment periods to avoid starting the intervention during summer break. Second, to sustain trust, we hired a CHW to address communication and scheduling concerns and maintain close contact with eligible participants. CHWs serve on the front lines of public health and are either deeply trusted by the communities they support or possess an intimate understanding of their needs and experiences (62). In BGM we incorporated the role of the CHW to enhance communication and provide social support from recruitment through start of intervention (63, 64). Examples of enhanced communication strategies included text message check-ins and referring participants to a BGM website with health information.

#### Changes in eligibility criteria and recruitment sites

Twenty one percent (*n* = 21) of the adolescent participants and their mothers attended baseline assessments and were found to be outside the initial study weight criteria, i.e., high-normal weight (between ≥50th and <85th percentile for age and gender) or overweight (between ≥85th and <95th percentile for age and gender). These adolescent participants were excluded from the study. In response, we requested and received IRB approval to amend the study protocol to also include daughters with underweight (<5th percentile) or obesity (≥95th percentile) based on BMI for age and gender. The daughters with underweight or obesity were required to provide a healthcare provider release to ensure connection to established care in case of an underlying metabolic disorder. Our modified weight criterion opened eligibility to adolescents with normal weight (≥5th and <85th percentile for age and gender) (65), if they reported either a poor diet (consuming <1 vegetable or <1 fruit per day) (66) or inadequate PA (completing <60 min per day of MVPA, 7 days per week).

**Figure 2:**
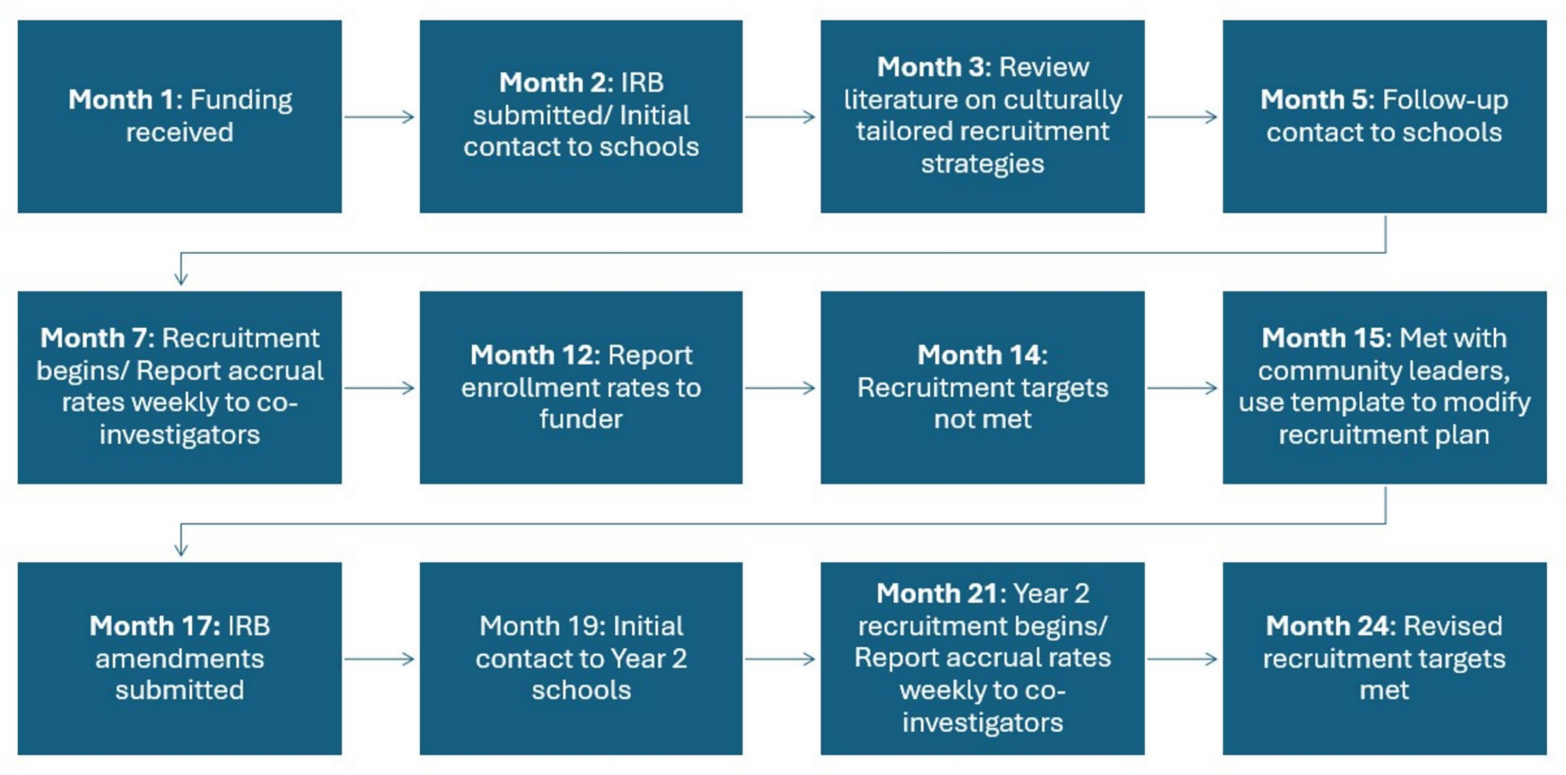
Timeline.

Due to the small number of daughters who initially met the inclusion criteria to participate in the study, it was determined that a wider pool of eligible daughters was needed to meet the study aims. Accordingly, we: (1) received IRB approval to amend the protocol to include 7^th^–8^th^ graders at least 13-years-old and (2) reduced the recruitment target for dyads from 24 dyads (12 dyads per school/per condition) to 6 dyads per school/per condition. If the total number of recruited dyads was less than 12 we would not cancel the school, but instead proceed with either an intervention or control group to assess feasibility. To ensure opportunity to evaluate the feasibility of the BGM intervention, schools with less than 12 recruited dyads were assigned to BGM until two cohorts received BGM. After that, schools will be assigned to DOCC until one cohort receives DOCC. Additional cohorts will be randomly assigned to BGM or DOCC. A timeline of recruitment events from study start to revision of recruitment targets is depicted in Figure 2. In Year 2, with the revised criteria, the BGM study successfully recruited and ran intervention groups with 10 daughter and mother dyads at one school and 7 dyads at another.

#### Competing demands influenced engagement

Competing demands resulted in last minute cancelations or no shows (67) negatively impacting accrual of Black adolescent daughter/mother dyads. Competing demands refer to other priorities that may compete with research participation, such as school, work, family commitments, and social activities (54). Needing to work, being a primary caretaker of children and/or relatives, being the single head of household, and time off work or travel costs related to participation presented conflicts to engaging in research activities (35, 68). To surmount multiple competing demands the following strategies were employed: (1) increased remuneration for time by increasing gift card incentives from $20 to $40 per participant for each data collection period, (2) hosted sessions and appointments at a convenient location in the community on weekends, (3) provided onsite childcare, (4) offered consent and assent via Zoom, (5) offered self-administered surveys via Research Electronic Data Capture (REDCap), and (6) conducted home visits to complete data collection assessments following common home visiting safety guidelines (69).

## Discussion

The purpose of this paper was to describe the lessons learned from 2 years of recruiting participants for BGM, an obesity prevention/weight maintenance intervention targeted and tailored for Black adolescent daughter/mother dyads. For recruitment, we learned that: (1) prior weight eligibility criteria were too prescriptive and (2) we needed to change recruitment targets to minimize school loss. For retention we learned that: (1) maintaining relationships with participants from screening to baseline and fostering consistent interactions throughout the study were essential and (2) competing demands influenced engagement.

BGM was designed to focus on obesity prevention/weight maintenance as opposed to weight loss or obesity treatment. Therefore, the original weight criteria excluded adolescent females with obesity to target individuals with high-normal weight or overweight. We revised our sampling criteria to include daughters with underweight (<5th percentile) or obesity (≥95th percentile) based on BMI for age and gender. For these individuals, it is important to frame strategies around weight maintenance rather than loss, as studies suggest lost weight is likely to be regained within 1–5 years (70, 71). Our revised sampling criteria were informed by Health at Every Size (HAES), a weight neutral approach that emphasizes health promoting behaviors rather than size or weight (72). The core principles are weight inclusivity, health enhancement, eating for wellbeing, life enhancing movement, and respectful care. A focus group of 41 Black women aged 18–24 assessed their perceptions of HAES and provided their insights in three themes: health is multidimensional, good health means “taking care of yourself,” and systemic and environmental disparities impact Black women’s health (72). While these perceptions of HAES principles among the young adults in the focus groups align with the BGM strategies of weight maintenance, the perceptions of HAES principles among Black adolescent daughter/mother dyads require investigation.

Although we incorporated strategies to *establish* trusting relationships for recruitment, we needed additional strategies to *maintain* trusting relationships for retention. Winter et al. (35), suggest minimizing duration of study components, such as assessment periods, as much as possible to enhance participant retention. Long gaps in engagement may increase dropout rates due to loss of participant interest and trust, or change in availability (35).

The CHW’s previous training gave her a comprehensive understanding of evidence-based health promotion strategies for Black adolescent and adult female populations. Based on her prior experience, the CHW suggested engaging participants through regular check-ins via text messaging and communicating relevant information via easily accessible and sustainable platforms such as the study website. Emerging data suggest that text messaging is associated with increased motivation, knowledge, and comfort in Black mothers participating in behavioral change clinical trials (73, 74). Additionally, using a website with study updates and health information are acceptable strategies to improve retention (60). We recommend that future studies systematically investigate these strategies to determine their efficacy.

## Conclusion and limitations of the study

Our experience delivering a race conscious, obesity prevention intervention for Black adolescent females and their mothers resulted in the identification of several recruitment challenges. Leveraging our already established partnerships with key leaders in the study schools, we collaboratively addressed recruitment challenges by implementing community informed strategies. The guidelines for evaluating the feasibility of recruitment in pilot studies of diverse populations and the Community-Informed Recruitment Plan template (i.e., template) both offered a framework for systematically evaluating and measuring recruitment and retention feasibility.

A limitation of this study is that we did not systematically incorporate the template into the initial development of the recruitment plan. Future studies should test the guidelines for evaluating the feasibility of recruitment and the template to increase recruitment and retention in a large-scale randomized control trial. We found that this culturally tailored, race-conscious approach was valuable in enhancing recruitment strategies. However, since we included several strategies, we did not have the capability of evaluating individual strategies to assess which of the changes was most effective. Finally, given the small sample size of this study, the findings cannot be generalized.

Justice in nursing ethics implores nurses to provide equitable treatment to all they serve. In the current research climate there are funding limitations, academic pressures, policy shifts, and possibly institutional barriers to implementing community engaged interventions. Despite these challenges, it is imperative to address health disparities while centering the experiences of marginalized populations. Now, more than ever, justice is needed in the conceptualization, design, implementation, and evaluation of health disparities interventions to achieve equitable health outcomes such as obesity prevention/weight maintenance projects for Black adolescent daughter/mother dyads.

## Data Availability

The original contributions presented in the study are included in the article/supplementary material, further inquiries can be directed to the corresponding author.
